# Dose prediction for repurposing nitazoxanide in SARS‐CoV‐2 treatment or chemoprophylaxis

**DOI:** 10.1111/bcp.14619

**Published:** 2020-12-01

**Authors:** Rajith K. R. Rajoli, Henry Pertinez, Usman Arshad, Helen Box, Lee Tatham, Paul Curley, Megan Neary, Joanne Sharp, Neill J. Liptrott, Anthony Valentijn, Christopher David, Steven P. Rannard, Ghaith Aljayyoussi, Shaun H. Pennington, Andrew Hill, Marta Boffito, Steve A. Ward, Saye H. Khoo, Patrick G. Bray, Paul M. O'Neill, W. David Hong, Giancarlo A. Biagini, Andrew Owen

**Affiliations:** ^1^ Department of Molecular and Clinical Pharmacology, Materials Innovation Factory University of Liverpool Liverpool UK; ^2^ Department of Chemistry University of Liverpool Liverpool UK; ^3^ Centre for Drugs and Diagnostics, and Department of Tropical Disease Biology Liverpool School of Tropical Medicine Liverpool UK; ^4^ Chelsea and Westminster NHS Foundation Trust and St Stephen's AIDS Trust 4th Floor Chelsea and Westminster Hospital London UK; ^5^ Jefferiss Research Trust Laboratories, Department of Medicine Imperial College London UK; ^6^ Pat Bray Electrical Orrell Wigan UK

**Keywords:** coronavirus, COVID‐19, lung, pharmacokinetics, SARS‐CoV‐2

## Abstract

**Background:**

Severe acute respiratory syndrome coronavirus 2 (SARS‐CoV‐2) has been declared a global pandemic and urgent treatment and prevention strategies are needed. Nitazoxanide, an anthelmintic drug, has been shown to exhibit in vitro activity against SARS‐CoV‐2. The present study used physiologically based pharmacokinetic (PBPK) modelling to inform optimal doses of nitazoxanide capable of maintaining plasma and lung tizoxanide exposures above the reported SARS‐CoV‐2 EC_90_.

**Methods:**

A whole‐body PBPK model was validated against available pharmacokinetic data for healthy individuals receiving single and multiple doses between 500 and 4000 mg with and without food. The validated model was used to predict doses expected to maintain tizoxanide plasma and lung concentrations above the EC_90_ in >90% of the simulated population. PopDes was used to estimate an optimal sparse sampling strategy for future clinical trials.

**Results:**

The PBPK model was successfully validated against the reported human pharmacokinetics. The model predicted optimal doses of 1200 mg QID, 1600 mg TID and 2900 mg BID in the fasted state and 700 mg QID, 900 mg TID and 1400 mg BID when given with food. For BID regimens an optimal sparse sampling strategy of 0.25, 1, 3 and 12 hours post dose was estimated.

**Conclusion:**

The PBPK model predicted tizoxanide concentrations within doses of nitazoxanide already given to humans previously. The reported dosing strategies provide a rational basis for design of clinical trials with nitazoxanide for the treatment or prevention of SARS‐CoV‐2 infection. A concordant higher dose of nitazoxanide is now planned for investigation in the seamless phase I/IIa AGILE trial.

**What is already known about this subject**

- COVID‐19, an acute respiratory infection caused by SARS‐CoV‐2, has been declared as a pandemic by the World Health Organization. Several repurposed drugs are being evaluated but currently there are no robustly validated treatment or preventative medicines or regimens. Nitazoxanide has shown in vitro activity against SARS‐CoV‐2, influenza and several other animal and human RNA viruses.

**What this study adds**

- The manuscript describes the pharmacokinetics of tizoxanide, the active metabolite of nitazoxanide using a physiologically based pharmacokinetic (PBPK) model. The validated PBPK model was used to estimate the optimal doses required for SARS‐CoV‐2 treatment or prevention such that >90% of the simulated population would have tizoxanide concentrations in the plasma and lung above the reported 90% effective concentration (EC_90_) value of nitazoxanide for the entire dosing interval.
- There are no reported studies that identify treatment regimens of nitazoxanide for SARS‐CoV‐2 treatment or prevention. The current study provides support for alternative dosing regimens of nitazoxanide for SARS‐CoV‐2 treatment and prevention that exceeds antiviral EC_90_ values in key tissues and organs for the duration of the dosing interval.
- Efficacy of nitazoxanide has been demonstrated for uncomplicated influenza and the presented predictions may help inform nitazoxanide dose selection for COVID‐19 clinical trials.

## 1 INTRODUCTION

COVID‐19 is a respiratory illness caused by severe acute respiratory syndrome coronavirus 2 (SARS‐CoV‐2) with noticeable symptoms such as fever, dry cough, and difficulty in breathing.^1^ There are currently no effective treatment or prevention options and it has become a global health problem with more than 3.1 million cases and over 217,000 deaths as of 29 April 2020.^2^ Urgent strategies are required to manage the pandemic and the repurposing of already approved medicines is likely to bring options forward more quickly than full development of potent and specific antivirals. Antiviral drugs may have application prior to or during early infection, but may be secondary to immunological interventions in later stages of severe disease.^3^

Although new chemical entities are likely to have high potency and specificity for SARS‐CoV‐2, full development is time‐consuming and costly, and attrition in drug development is high.^4^, ^5^ Drug repurposing, where existing or investigational drugs could be used outside the scope of their original indication, may present a rapid alternative to new drug development. Several examples of successful repurposing exist, including the use of the anti‐angiogenic drug thalidomide for cancer, the use of mifepristone for Cushing's disease after initially being approved for termination of early pregnancy and the repurposing of sildenafil from angina to erectile disfunction.^6^, ^7^ It should be noted, however, that drug repurposing is considerably faster when the approved dose is successfully repurposed, with additional complexity in clinical development when higher doses are required.

SARS‐CoV‐2 targets the angiotensin‐converting enzyme 2 (ACE2) receptors that are present in high density on the outer surface of lung cells.^1^ Lungs are the primary site of SARS‐CoV‐2 replication and infection is usually initiated in the upper respiratory tract.^8^ Symptoms that result in neurological, renal and hepatic dysfunction are also emerging due to the expression of ACE2 receptors in these organs.^9^, ^10^, ^11^, ^12^ Therefore, therapeutic concentrations of antiviral drugs are likely to be needed in the upper airways for treatment and prevention of infection, but sufficient concentrations are also likely to be required systemically for therapy to target the virus in other organs and tissues.

The scale at which the antiviral activity of existing medicines is being studied for potential repurposing against SARS‐CoV‐2 is unprecedented.^13^ The authors recently reported an analysis which benchmarked reported in vitro activity of tested drugs against previously published pharmacokinetic exposures achievable with their licenced doses.^14^ Importantly, this analysis demonstrated that the majority of drugs that have been studied for anti‐SARS‐CoV‐2 activity are unlikely to achieve the necessary concentrations in the plasma after administration of their approved doses. While this analysis is highly influenced by the drugs selected for analysis to date and highly sensitive to the accuracy of the reported antiviral activity data, a number of candidate agents were identified with plasma exposures above the reported EC_50_/EC_90_ against SARS‐CoV‐2.

One such drug, nitazoxanide, is a thiazolide antiparasitic medicine used for the treatment of cryptosporidiosis and giardiasis that cause diarrhoea,^15^, ^16^ and also has reported activity against anaerobic bacteria, protozoa and other viruses.^17^ Several reports have confirmed the activity of nitazoxanide against SARS‐CoV‐2 in different cell types.^18^, ^19^, ^20^, ^21^ Importantly, rapid deacetylation of nitazoxanide in blood means that the major systemic species of the drug in vivo is tizoxanide, which has been shown to exhibit similar in vitro inhibitory activity to nitazoxanide for rotaviruses,^22^ hepatitis B and C viruses,^23^, ^24^ other coronaviruses, noroviruses,^25^influenza viruses and SARS‐CoV‐2.^20^, ^26^, ^27^, ^28^ As another respiratory virus, previous work on influenza may be useful to gain insight into the expected impact of nitazoxanide for SARS‐CoV‐2. Accordingly, the drug has been shown to selectively block the maturation of the influenza haemagglutinin glycoprotein at the post‐translational stage^27^, ^29^ and a previous phase 2b/3 trial demonstrated a reduction in symptoms and viral shedding at a dose of 600 mg BID compared to placebo in patients with uncomplicated influenza.^30^ Other potential benefits of nitazoxanide in COVID‐19 may derive from its impact upon the innate immune response that potentiates the production of type 1 interferons ^27^, ^31^ and bronchodilation of the airways through inhibition of TMEM16A ion channels.^32^ As of 9 August 2020, a total of 19 trials were listed as either planned or recruiting on **clinicaltrials.gov** but all of these studies are focusing on doses of ≤1000 mg BID nitazoxanide either alone or in combination with\ other agents.^33^ However, there are currently no data within the public domain to support these doses for COVID‐19. Nitazoxanide is relatively safe in humans and a review of the safety and minimum pricing was recently published.^34^ Plasma concentrations of tizoxanide have demonstrated dose proportionality, but administration in the fed state increases the plasma exposure.^35^ Thus, the drug is recommended for administration with food.

The prerequisites for successful development of antiviral drugs for SARS‐CoV‐2 have yet to be elucidated and gaps in knowledge exist in terms of the exposure‐response relationship. However, the lung has emerged as a clear site of primary infection, and pulmonary co‐morbidities are a key driver of mortality in the sickest patients.^36^, ^37^, ^38^ Therefore, in treatment of early disease at least it seems likely that successful antiviral regimens will require drugs to penetrate into the lung at sufficient concentrations to exert their activity. Using HIV as a paradigm for successful chemoprophylactic approaches, antiviral drugs also require penetration into key sites of transmission such as the anal and vaginal mucosa.^39^, ^40^, ^41^ Therefore, the authors hypothesise that drugs achieving concentrations in lung that exceed those needed for activity will underpin successful antiviral development.

Physiologically based pharmacokinetic (PBPK) modelling is a computational tool that integrates human physiology and drug disposition kinetics using mathematical equations to inform the pharmacokinetic exposure using in vitro and drug physicochemical data.^42^ Recently, several international groups have called for a more robust integration of clinical pharmacology principles into COVID‐19 drug development.^43^, ^44^ Accordingly, the aim of this study was to validate a PBPK model for tizoxanide following administration of nitazoxanide. Once validated, this model was first used to assess the plasma and lung exposures estimated to be achieved during a previous trial for uncomplicated influenza. Next, different nitazoxanide doses and schedules were simulated to identify those expected to provide tizoxanide plasma and lung trough concentrations (*C*_trough_) above the reported nitazoxanide SARS‐CoV‐2 EC_90_ in the majority (>90%) of patients.

## 2 METHODS

A previously published whole‐body PBPK model consisting of compartments to represent select organs and tissues developed in Simbiology (MATLAB R2019a, MathWorks Inc., Natick, MA, USA) was used in this study.^45^, ^46^ Nitazoxanide physiochemical and drug‐specific parameters used in the PBPK model were obtained from literature sources as outlined in Table 1. The PBPK model was assumed to be blood‐flow limited, with instant and uniform distribution in each tissue or organ and no reabsorption from the large intestine. Since the data are computer generated, no ethics approval was required for this study.

**TABLE 1:** Nitazoxanide input parameters for the PBPK model

Parameter	Nitazoxanide	Tizoxanide
Molecular weight	307.28^[^ ^15^ ^]^	265.25^[^ ^56^ ^]^
Protein binding^a^	>99%^[^ ^15^ ^]^	>99%^[^ ^53^ ^]^
Log P	1.63^[^ ^15^ ^]^	3.2^[^ ^56^ ^]^
p*K*a (acidic)	8.3^[^ ^15^ ^]^	6.7^[^ ^57^ ^]^
*R*	0.55	0.55
Number of hydrogen bond donors	1^[^ ^15^ ^]^	2^[^ ^56^ ^]^
Polar surface area	114.11^[^ ^15^ ^]^	136^[^ ^56^ ^]^
Apparent permeability (cm/s)	1.11e‐4^[^ ^58^ ^]^	…
Apparent clearance (L/h)	…	19.34 ± 4.97^[^ ^55^ ^]^
Volume of distribution (L)	…	38.68 ± 14.02^[^ ^55^ ^]^
Half‐life (h)	…	1.38 ± 0.29^[^ ^55^ ^]^

*Note*. R, blood to plasma ratio was predicted from Paixão et al.^35^ not available, apparent permeability was assessed in HT29‐19A cells and this value was considered the same in caco‐2 cells for tizoxanide.

^a^ Protein binding was considered as 99% for the PBPK model.

### 2.1 Model development

One hundred virtual healthy adults (50% women, aged 20‐60 years between 40 and 120 kg) were simulated. The required duration for successful SARS‐CoV‐2 antiviral activity has not yet been robustly elucidated but for clarity in presentation, the simulations were conducted over 5 days of dosing. It should be noted that similar exposures would be expected beyond this once the drug has reached steady‐state pharmacokinetics. Patient demographics such as weight, body mass index and height were obtained from CDC charts.^47^ Organ weight/volumes and blood flow rates in humans were obtained from published literature sources.^48^, ^49^ Transit from the stomach and small intestine was divided into seven compartments to capture effective absorption kinetics as previously described.^50^ Tissue to plasma partition ratio of drug and drug disposition across various tissues and organs were described using published mathematical equations.^51^, ^52^, ^53^ Effective permeability (*P*_eff_) in humans was scaled from apparent permeability (*P*_app_) in HT29‐19A cells (due to lack of available data, it was assumed the same in Caco‐2 cells) using the following equations to compute the rate of absorption (*K*_a_ in h^−1^) from the small intestine.^54^, ^55^

$$\log_{10}P_{\mathrm{eff}}=0.6836\times \log_{10}P_{\mathrm{app}}-0.5579$$

$$K_{a}=\frac{2\times P_{\mathrm{eff}}\times 60\times 60}{r}$$

### 2.2 Model validation

The PBPK model was validated against available clinical data in healthy individuals in the fed and fasted state for various single oral doses of nitazoxanide ranging from 500 to 4000 mg,^35^, ^56^ and for multiple dosing at 500 and 1000 mg BID with food. Nitazoxanide absorption was considered using the available apparent permeability data (shown in Table 1) and tizoxanide was assumed to form as soon as the drug reached systemic circulation as metabolic studies have shown it takes just 6 minutes for complete conversion into the active circulating metabolite, with no trace of nitazoxanide detected in plasma.^57^ Therefore, tizoxanide parameters were used to define drug disposition. The elimination pathway of tizoxanide is not clear from the literature, therefore apparent clearance obtained from the literature was used as a first‐order rate from the veinal compartment. Due to the unavailability of transporter pathways, a fixed absorption rate computed from apparent permeability was considered in the model. The model was assumed to be validated if: (a) the absolute average fold error (AAFE) between the observed and the simulated plasma concentrations‐time curve of tizoxanide was less than two; and (b) the simulated pharmacokinetic parameters‐maximum concentration (*C*_max_), area under the plasma concentration‐time curve (AUC) and *C*_trough_ (trough concentration at the end of the dosing interval) were less than 2‐fold from the mean observed values.

### 2.3 Model simulations

The pharmacokinetics following administration of 600 mg BID as reported in a previous phase 2b/3 clinical trial of nitazoxanide in uncomplicated influenza^30^ were first simulated and plotted relative to the average of previously reported influenza EC_90_s^58^, ^59^ for strains (as shown in Supporting Information Table S1) included in the previous trials. This was done to assess the exposure relative to in vitro activity for an indication where clinical benefit was already demonstrated.

For potential SARS‐CoV‐2 applications, several oral dosing regimens were simulated for BID, TID or QID administration in the fasted state. Antiviral activity data from Wang et al^18^ were digitised using Web Plot Digitiser® software and used to calculate a nitazoxanide EC_90_ for SARS‐CoV‐2 of 4.64 μM (1.43 mg/L). Optimal doses were identified such that the concentration at 12 hours post first dose (*C*_12_) for BID, 8 hours post first dose (*C*_8_) for TID or 6 hours post first dose (*C*_6_) for QID administration were over the recalculated EC_90_ for nitazoxanide. Plasma and lung tizoxanide exposures at these doses and schedules are reported in addition to plasma‐time curves. The doses were optimised using tizoxanide parameters and pharmacokinetics, but the doses were reported for nitazoxanide.

### 2.4 Optimal pharmacokinetic sampling

Clinical trials should incorporate pharmacokinetic sampling to confirm tizoxanide plasma exposures and further validate the predictions from the PBPK model. Optimal sparse pharmacokinetic timepoint selection (assuming four blood samples per patient and 40 patients in the study) was made on the basis of the prior fed pharmacokinetic data of Stockis et al.^35^, ^56^ Tizoxanide plasma pharmacokinetic data in fed patients from Stockis et al was fitted with an empirical one‐compartment disposition model, with first‐order absorption and absorption transit compartment, and the parameters from this fitting were used (with nominal %CV interindividual variability in the pharmacokinetic parameters of 30%) in the optimal design software PopDes (University of Manchester Version 4.0) to generate the suggested optimal sampling timepoints.^60^, ^61^

### 2.5 Nomenclature of targets and ligands

Key protein targets and ligands in this article are hyperlinked to corresponding entries in http://www.guidetopharmacology.org, the common portal for data from the IUPHAR/BPS Guide to PHARMACOLOGY, and are permanently archived in the Concise Guide to PHARMACOLOGY 2019/20.

## 3 RESULTS

### 3.1 Model validation

The PBPK model validation against various fasted oral doses is shown in Supporting Information Figure S1 and the validation against single and multiple doses in the fed state is shown in Supporting Information Figure S2. The corresponding pharmacokinetic parameters (AUC, *C*_max_ and *C*_trough_) are presented in Tables 2 and 3. The AAFE values for the validated doses ranged between 1.01 and 1.55 for the fasted state and between 1.1 and 1.58 for the fed state, indicating a close match between observed and simulated data. The ratio between the simulated and the observed pharmacokinetic parameters (AUC, *C*_max_ and *C*_trough_) was between 0.81 and 1.54 (Table 2) for the fasted state and between 0.67 and 2.15 for the fed state. The PBPK model simulated tizoxanide plasma concentrations were within acceptable ranges and therefore the PBPK model was assumed to be validated.

**TABLE 2:** Tizoxanide validation against observed data for various single oral doses in the fasted state

Dose (mg)	Pharmacokinetic parameter	Observed	Simulated	Simulated/observed
500^39^	AUC_0‐12h_ (μg·h/mL)^a^	27.02 ± 6.24	24.32 ± 5.07	0.90
	*C*_max_ (μg/mL)^a^	6.80 ± 1.32	6.47 ± 1.17	0.95
	*C*_trough_ (μg/mL)^b^	0.106	0.119 ± 0.185	1.12
1000^29^	AUC_0‐24h_ (μg·h/mL)^c^	50.6 (29.0‐88.4)	48.9 (34.3‐63.4)	0.93
	*C*_max_ (μg/mL)^c^	12.3 (8.21‐18.3)	12.9 (10.2‐12.9)	1.05
	*C*_trough_ (μg/mL)^b^	0.23	0.21 (0.09‐0.53)	0.93
2000^29^	AUC_0‐24h_ (μg·h/mL)^c^	59.2 (36.5‐95.9)	68.5 (48.9‐84.1)	1.12
	*C*_max_ (μg/mL)^c^	9.08 (7.07‐11.7)	9.45 (7.18‐11.7)	1.04
	*C*_trough_ (μg/mL)^b^ ^,^ ^c^	1.49	1.33 (0.45‐2.21)	0.89
3000^29^	AUC_0‐24h_ (μg·h/mL)^c^	52.9 (34.6‐81.0)	42. 6 (31.7‐53.6)	0.81
	*C*_max_ (μg/mL)^c^	7.39 (6.07‐8.98)	7.51 (5.68‐9.33)	1.02
	*C*_trough_ (μg/mL)^b^ ^,^ ^c^	0.6	0.93 (0.79‐1.06)	1.54
4000^29^	AUC_0‐24h_ (μg·h/mL)^c^	88.5 (53.5‐146)	76.9 (60.1‐93.8)	0.87
	*C*_max_ (μg/mL)^c^	10.5 (8.16‐13.5)	9.58 (7.34‐11.8)	0.91
	*C*_trough_ (μg/mL)^b^ ^,^ ^c^	2.48	2.44 (2.19‐2.67)	0.98

^a^ *C*_max_ and AUC_0‐12h_ are represented as arithmetic mean ± SD.

^b^ *C*_trough_ is *C*_12_ and has been digitised from the pharmacokinetic curve as the geometric mean is not available. The arithmetic mean is shown for observed and arithmetic mean (mean – SD, mean + SD) is shown for simulated data.

^c^ *C*_max_ and AUC_0‐24h_ are represented as geometric mean (mean – SD, mean + SD). *C*_max_, AUC_0‐24h_ and *C*_trough_ were normalised to a 1000 mg dose.

**TABLE 3:** Tizoxanide validation against observed data for various single and multiple oral doses when given with food

Dose (mg)	Pharmacokinetic parameter	Observed	Simulated	Simulated/observed
500 (single)^53^	AUC_0‐last_ (μg·h/mL)^a^	41.8 (36.1‐48.4)	33.2 (23.1‐43.2)	0.79
	*C*_max_ (μg/mL)	10.4 (8.65‐12.6)	8.08 (6.17‐9.98)	0.78
	*C*_trough_ (μg/mL)^b^	0.178	0.28 (0‐0.5)	1.56
500 BID (multiple)^53^	AUC_0‐12h_ (μg·h/mL)^a^	48.7 (36.0‐65.9)	32.8 (19.5‐46.1)	0.67
	*C*_max_ (μg/mL)	9.05 (7.13‐11.5)	7.66 (5.89‐9.44)	0.85
	*C*_trough_ (μg/mL)^b^	0.310	0.35 (0‐0.89)	1.11
1000 (single)^53^	AUC_0‐last_ (μg·h/mL)^a^	85.9 (68.8‐107)	91.3 (69‐113.5)	1.06
	*C*_max_ (μg/mL)	14.4 (10.8‐19.2)	17.8 (14.3‐21.3)	1.23
	*C*_trough_ (μg/mL)^b^	0.786	1.43 (0.2‐2.22)	1.82
1000 BID (multiple)^53^	AUC_0‐12h_ (μg·h/mL)^a^	144 (105‐198)	90.9 (69‐112.8)	0.63
	*C*_max_ (μg/mL)	24.2 (20.3‐28.7)	18.2 (14.4‐21.9)	0.75
	*C*_trough_ (μg/mL)^b^	1.68	1.38 (0.33‐2.07)	0.82
2000 (single) ^29^	AUC_0‐24h_ (μg·h/mL)^c^	110 (88.0‐139)	109 (74.8‐142)	0.99
	*C*_max_ (μg/mL)^c^	15.8 (13.0‐19.2)	14.3 (11.2‐17.4)	0.91
	*C*_trough_ (μg/mL)^b^ ^,^ ^c^	1.52	3.27 (1.73‐4.46)	2.15
3000 (single) ^29^	AUC_0‐24h_ (μg·h/mL)^c^	95.3 (60.0‐152)	90 (60.9‐119)	0.94
	*C*_max_ (μg/mL)^c^	10.0 (7.40‐13.5)	10.4 (8.09‐12.7)	1.04
	*C*_trough_ (μg/mL)^b^ ^,^ ^c^	2.35	2.64 (1.44‐3.56)	1.12
4000 (single) ^29^	AUC_0‐24h_ (μg·h/mL)^c^	192 (99.5‐370)	161 (125‐196)	0.84
	*C*_max_ (μg/mL)^c^	17.5 (11.5‐26.5)	14.2 (11.2‐17.2)	0.81
	*C*_trough_ (μg/mL)^b^ ^,^ ^c^	6.55	6.52 (5.04‐7.84)	0.99

^a^ AUC is represented as AUC_0‐∞_ after the first dose for single and AUC_0‐12_ on day 7.

^b^ *C*_trough_ is *C*_12_ and has been digitised from the pharmacokinetic curve as the geometric mean is not available. The arithmetic mean is shown for observed and arithmetic mean (mean – SD, mean + SD) is shown for simulated data.

^c^ *C*_max_ and AUC are represented as geometric mean (mean – SD, mean + SD) and *C*_max_ and AUC_0‐24h_ were normalised to a 1000 mg dose.

### 3.2 Model simulations

**FIGURE 1:**
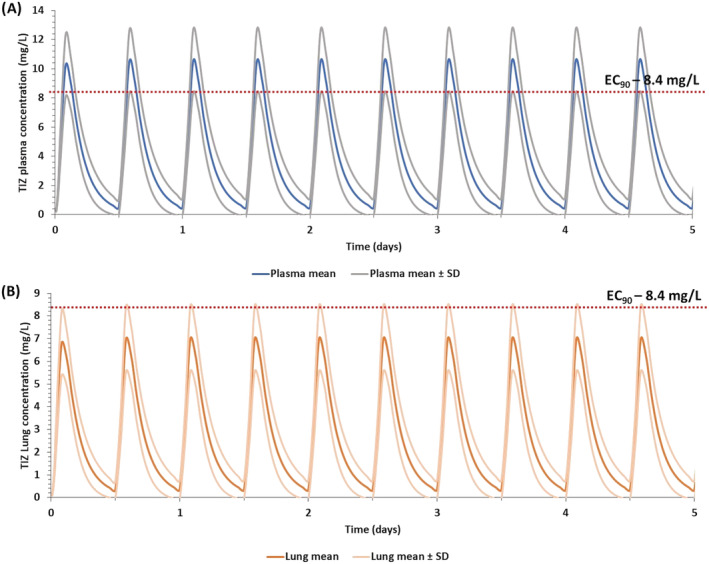
Simulated plasma A, and lung B, concentrations for nitazoxanide 600 mg BID for 5 days with food relative to the average reported tizoxanide EC_90_ value for influenza strains (Supporting Information Table S1) similar to those in a previous phase 2b/3 trial in uncomplicated influenza^30^

Figure 1a,b shows the simulated plasma and lung exposures relative to the average influenza EC_90_ after administration of 600 mg BID dose of nitazoxanide with food as reported in the previous phase 2b/3 trial in uncomplicated influenza.^30^ These simulations indicate that all patients were predicted to have plasma and lung tizoxanide *C*_trough_ (*C*_12_) concentrations below the average EC_90_ (8.4 mg/L, Supporting Information Table S1),^59^ but that 71% and 14% were predicted to have plasma and lung *C*_max_ concentrations, respectively, above the average EC_90_ for influenza, respectively.

**FIGURE 2:**
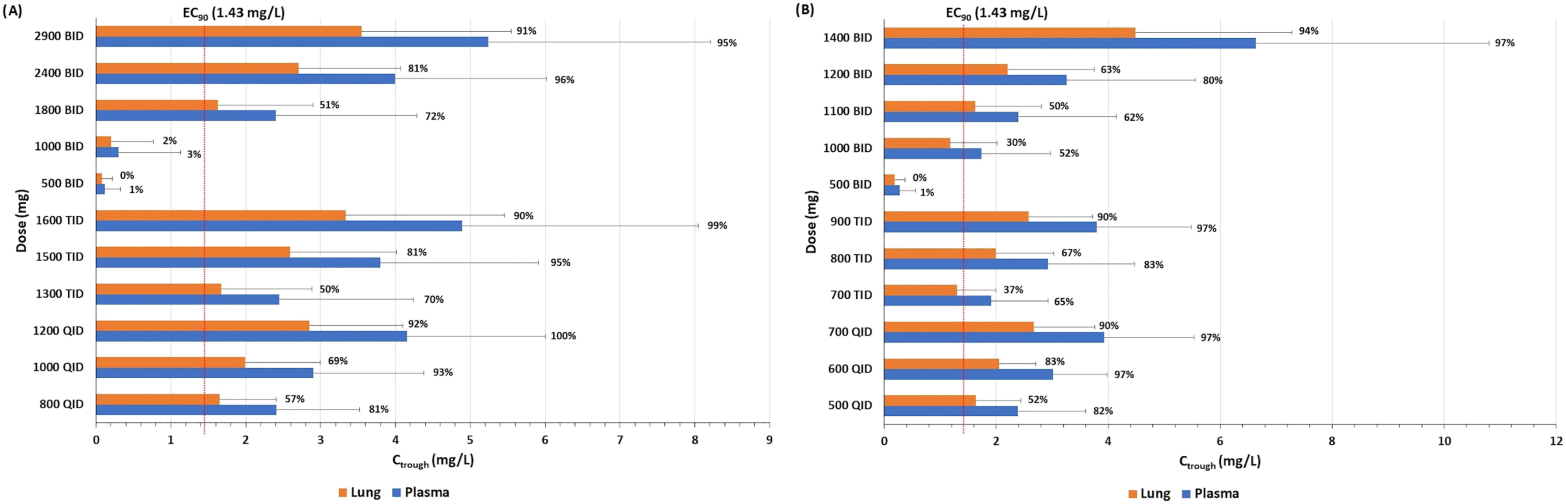
A, Predicted tizoxanide *C*_trough_ (BID 12 hours, TID 8 hours, QID 6 hours) for various dosing regimens of nitazoxanide in the fasted state at the end of the first dose. Data are presented as means and error bars represent standard deviation. The percentages adjacent to the bar chart indicate the percentages of simulated population over EC_90_ of nitazoxanide for SARS‐CoV‐2. B, Predicted tizoxanide *C*_trough_ (BID 12 hours, TID 8 hours, QID 6 hours) for various dosing regimens of nitazoxanide in the fed state at the end of the first dose. Data are presented as means and error bars represent standard deviation. The percentages adjacent to the bar chart indicate the percentages of simulated population over EC_90_ of nitazoxanide for SARS‐CoV‐2

**FIGURE 3:**
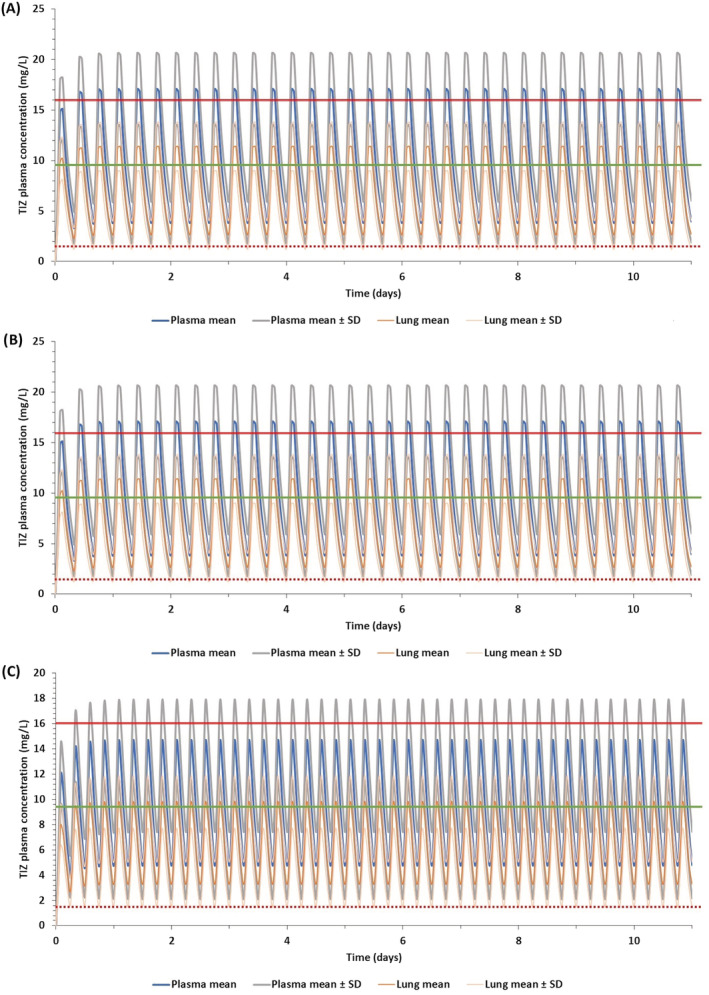
Predicted plasma and lung concentrations for optimal doses during the fed state at different regimens reaching steady state: A, 1400 mg BID, B, 900 mg TID and C, 700 mg QID. TIZ, tizoxanide; SD, standard deviation. The solid red line indicates clinical *C*_max_ of a 1 g single dose at the fed state,^35^ the solid green line represents clinical *C*_max_ of a 500 mg single dose^62^ at the fed state and the dotted red line represents the EC_90_ of nitazoxanide for SARS‐CoV‐2^18^

Figure 2a,b shows the prediction of trough concentrations in plasma and lung for the different simulated doses and schedules in healthy individuals for fasted and fed states. Doses and schedules estimated to provide plasma *C*_trough_ concentrations over 1.43 mg/L for at least 50% of the simulated population were identified. However, lower doses in each schedule (ie, 800 mg QID, 1300 mg TID and 1800 mg BID in the fasted state and 500 mg QID, 700 mg TID and 1100 mg BID in the fed state) were predicted to result in >40% of the simulated population having lung *C*_trough_ below the SARS‐CoV‐2 EC_90_. Optimal doses for SARS‐CoV‐2 in the fasted state were predicted to be 1200 mg QID, 1600 mg TID and 2900 mg BID, and in the fed state were 700 mg QID, 900 mg TID and 1400 mg BID. Figure 3 shows the plasma and lung concentrations for the optimal doses and schedules in the fed state and Supporting Information Figure S3 shows the plasma concentration‐time profile of optimal doses in the fasted state. Tizoxanide concentrations in lung and plasma were predicted to reach steady state in <48 hours, in both the fasted and fed stated.

### 3.3 Optimal sparse sampling design

Results from PopDes optimal design procedure indicate pharmacokinetic sampling timepoints at 0.25, 1, 3 and 12 hours post dose for BID regimens, and 0.25, 1, 2 and 8 hours post dose for TID regimens.

## 4 DISCUSSION

Treatment of SARS‐CoV‐2 has become a major global healthcare challenge, with no well‐defined therapeutic agents to either treat or prevent the spread of the infection. Short‐term treatment options are urgently required but many ongoing trials are not based on a rational selection of candidates in the context of safe achievable drug exposures. In the absence of a vaccine, there is also an urgent need for chemopreventative strategies to protect those at high risk such as healthcare staff, key workers and household contacts who are more vulnerable to infection. Nitazoxanide has emerged as a potential candidate for repurposing for COVID‐19. The PBPK model presented herein was validated with an acceptable variation in AAFE and simulated/observed ratio (close to 1), which provides confidence in the presented predictions. The present study aimed to define the optimal doses and schedules for maintaining tizoxanide plasma and lung concentrations above the reported nitazoxanide EC_90_ for the duration of the dosing interval.

Nitazoxanide was assessed in a double‐blind, randomised, placebo‐controlled, phase 2b/3 trial (NCT01227421) of uncomplicated influenza in 74 primary care clinics in the United States between 27 December 2010 and 30 April 2011.^30^ The median duration of symptoms for patients receiving placebo was 117 hours compared with 96 hours in patients receiving 600 mg BID nitazoxanide with food. Importantly, virus titre in nasopharyngeal swabs in 39 patients receiving nitazoxanide 600 mg BID was also lower than in 41 patients receiving placebo. The average of reported tizoxanide EC_90_s for influenza A and B^63^ was calculated to be 8.4 mg/L, which is higher than that reported EC_90_ for nitazoxanide against SARS‐CoV‐2.^18^ The PBPK model was used to simulate plasma and lung exposures after administration of 600 mg BID for 5 days, and while plasma *C*_max_ exceeded the average influenza EC_90_ in the majority of patients, the *C*_trough_ values did not. The modelling data suggest that the moderate effects of nitazoxanide seen in influenza could be a function of underdosing. Although influenza and SARS‐CoV‐2 both elicit pulmonary disease, it should be noted that the viruses are quite dissimilar. However, taken collectively when benchmarked against the in vitro activities of both viruses, these data are encouraging for the application of nitazoxanide in COVID‐19. Moreover, these simulations indicate that higher doses may be optimal for maximal suppression of other pulmonary viruses.

In some cases, food intake may be difficult in patients with COVID‐19 so drugs that can be given without regard for food may be preferred. However, the presented predictions indicate that optimal plasma and lung exposures would require 1200 mg QID, 1600 mg TID or 2900 mg BID in the fasted state. Conversely, the PBPK models predict that doses of 700 mg QID, 900 mg TID or 1400 mg BID with food provide tizoxanide concentrations in plasma and lung above the EC_90_ value for nitazoxanide for the entire dosing interval in at least 90% of the simulated population. Single doses up to 4000 mg have been administered to humans previously^35^ but the drug is usually administered at 500 mg BID. The PBPK model simulations indicate a high BID dose of 1400 mg (fed) and caution may be needed for gastrointestinal intolerance at this dose. The simulations indicate that lower TID and QID dosing regimens may also warrant investigation, and 900 mg TID as well as 700 mg QID (both with food) regimens are also predicted to provide optimal exposures for efficacy. Importantly, the overall daily dose was estimated to be comparable between the different optimal schedules and it is unclear whether splitting the dose will provide gastrointestinal benefits. For prevention application where individuals will need to adhere to regimens for longer durations, minimising the frequency of dosing is likely to provide adherence benefits. However, for short‐term application in therapy, more frequent dosing may be more acceptable to minimise gastrointestinal intolerance.

The nitazoxanide mechanism of action for SARS‐CoV‐2 is currently unknown. However, for influenza it has been reported to involve interference with *N*‐glycosylation of haemagglutinin.^27^, ^63^, ^64^ Since the SARS‐CoV‐2 spike protein is also heavily glycosylated^65^ with similar cellular targets in the upper respiratory tract, a similar mechanism of action may be expected.^8^, ^66^ An ongoing trial in Mexico is being conducted with 500 mg BID nitazoxanide with food,^33^ but these doses may not be completely optimal for virus suppression across the entire dosing interval.

This analysis provides a rational dose optimisation for nitazoxanide for treatment and prevention of COVID‐19. However, there are some important limitations that must be considered. PBPK models can be useful in dose prediction but the quality of predictions is only as good as the quality of the available data on which they are based. Furthermore, the mechanism of action for nitazoxanide for other viruses has also been postulated to involve an indirect mechanism through amplification of the host innate immune response,^67^ and this would not have been captured in the in vitro antiviral activity that informed the target concentrations for this dose prediction. The simulated population used in this modelling consisted of healthy individuals up to 60 years old, but many patients requiring therapy may be older and have underlying comorbidities. To the best of our knowledge, the impact of renal and hepatic impairment on pharmacokinetics of this drug have not been assessed and may impact the pharmacokinetics. Although the current PBPK model is validated against various single doses in the fasted state and a few multiple doses when given with food, the model may predict with less accuracy for multiple doses due to the unavailability of clinical data for multiple dosing over 1000 mg. The unusual shape of the observed PK curve in Supporting Information Figure S1e (4000 mg, fasted state) and Supporting information Figure S2d (3000 mg, fed state) may be an error in the sampling or due to the low number of individuals in the study and this cannot be captured using the PBPK model since the exact cause of this is unknown, but the AAFE values and the ratio between observed and simulated PK parameters is less than 2, which implies the model as validated. Also, the observed versus simulated curve for preceding doses, ie, 3000 mg in the fasted state (Supporting Information Figure S1d) and 2000 mg in the fed state (Supporting Information Figure S2c) are similar and the informed optimal doses are below these doses (2900 mg fasted and 1400 mg fed), therefore the predictions would not have any impact of deviations in this case. The presented models were validated using BID doses only, and confidence in the predictions for TID and QID doses may be lower. The clinical studies used for model validation were performed in a limited number of patients^35^ and thus may underrepresent real intersubject variability. Also, the disposition parameters (apparent clearance and rate of absorption) obtained for the PBPK model were from a fasted study of 500 mg BID and the parameters were adjusted to validate the tizoxanide model in the fed state, which may limit confidence in the model at higher doses. Only one manuscript has described the in vitro activity of nitazoxanide against SARS‐CoV‐2^18^ and no data are available for tizoxanide. Reported in vitro data may vary across laboratories and due to this the predicted optimal doses may change. However, the reported comparable activity of nitazoxanide and tizoxanide against a variety of other viruses (including other coronaviruses) does strengthen the rationale for investigating this drug for COVID‐19.^22^, ^23^, ^24^, ^26^, ^27^ Recently, several international investigators with experience of protein binding and its application in successful therapy for other viruses initiated a discussion about protein binding to reach consensus on its correct interpretation for SARS‐CoV‐2.^68^ As an outcome of this consensus, care should be taken to neither over‐ nor under‐represent its consequences. Unfortunately, assessment of the consequences of protein binding needs to involve empirical determination as part of the in vitro methodology and none of the reported EC_90_ values for influenza or SARS‐CoV‐2 were protein binding‐adjusted.^18^ Tizoxanide is known to be highly protein bound (>99%) in plasma,^69^ but while this was used to estimate drug penetration into the lung, data were not available to correct the in vitro activity to make a robust assessment in relation to the free drug pharmacokinetics.

The doses estimated to be necessary to maintain active tizoxanide concentrations in plasma and lung are considerably higher than the approved dose (500 mg BID) or other multiple dose studies that have been published to date. However, single doses of up to 4000 mg have been given safely to humans previously, and several of the authors recently reviewed the safety of nitazoxanide across the different doses at which it has been studied.^34^, ^35^ Nitazoxanide appears to be a remarkably safe drug but the major concerns are likely to relate to gastrointestinal safety. Accordingly, the doses proposed here will require a clinical development pathway that robustly addresses safety. As a first step, Unitaid have recently agreed funding and an independent scientific advisory board has approved inclusion of high‐dose nitazoxanide in the seamless phase I/IIa AGILE platform trial (www.agiletrial.net), subject to successful relevant ethical and regulatory approvals.

In summary, the developed PBPK model of nitazoxanide was successfully validated against clinical data and based on currently available data optimal doses for COVID‐19 were estimated to be 700 mg QID, 900 mg TID or 1400 mg BID with food. Should nitazoxanide be progressed into clinical evaluation for treatment and prevention of COVID‐19, it will be important to further evaluate the pharmacokinetics in these population groups. In treatment trials particularly, intensive pharmacokinetic sampling may be challenging. Therefore, an optimal sparse sampling strategy for BID, TID and QID dosing is also presented.

## COMPETING INTERESTS

A.O. and S.P.R. are Directors of Tandem Nano Ltd. A.O. has received research funding from ViiV, Merck, Janssen and consultancy from Gilead, ViiV and Merck not related to the current paper. P.O.N. is currently engaged in a collaboration with Romark LLC but this interaction did not influence the prioritisation or conclusions in the current manuscript. No other conflicts are declared by the authors.

## CONTRIBUTORS

All authors contributed intellectually to the work during the UK COVID19 lockdown and commented on multiple drafts of the manuscript. A.O. and R.R. designed the research. U.A., H.B., L.T., H.P. and A.O. performed the research. R.R. and H.P. analysed the data.

## Supporting information


**Table S1** Nitazoxanide 50% and 90% effective concentrations against various influenza virus strains
**Figure S1** Comparison of simulated and observed plasma concentration‐time curve of tizoxanide (TIZ) at fasted state: (A) 500 mg, (B) 1000 mg, (C) 2000 mg, (D) 3000 mg and (E) 4000 mg
**Figure S2** Comparison of simulated and observed plasma concentration‐time curve of tizoxanide (TIZ) for single and multiple dosing regimen (where available) at fed state: (A) 500 mg, (B) 1000 mg, (C) 2000 mg, (D) 3000 mg and (E) 4000 mg
**Figure S3** Predicted plasma and lung concentrations for optimal doses during the fed state at different regimens reaching steady state ‐ (A) 1400 mg BID, (B) 900 mg TID and (C) 700 mg QID. TIZ, tizoxanide, SD, standard deviation, solid red line indicates clinical *C*_max_ of 1 g single dose at fed state [35], solid green line represents clinical *C*_max_ of 500 mg single dose [70] at fed state and the dotted red line represents the EC_90_ of nitazoxanide for SARS‐CoV‐2 [18]

## Data Availability

The data presented in this study is available from the corresponding author upon request.

## References

[1] Machhi J , Herskovitz J , Senan AM , et al. The natural history, pathobiology, and clinical manifestations of SARS‐CoV‐2 infections. J Neuroimmune Pharmacol. 2020;15(3):359‐386. 32696264 10.1007/s11481-020-09944-5 PMC7373339

[2] Johns Hopkins University of Medicine . *Coronavirus Resource Center*. 2020 [cited 2020 17/04/2020]; Available from: https://coronavirus.jhu.edu/map.html

[3] Zhang W , Zhao Y , Zhang F , et al. The use of anti‐inflammatory drugs in the treatment of people with severe coronavirus disease 2019 (COVID‐19): the perspectives of clinical immunologists from China. Clin Immunol (Orlando, Fla). 2020, 214;108393‐108393. 10.1016/j.clim.2020.108393 PMC7102614 32222466

[4] Gns HS , Gr S , Murahari M , Krishnamurthy M . An update on drug repurposing: re‐written saga of the drug's fate. Biomed Pharmacother. 2019;110:700‐716. 30553197 10.1016/j.biopha.2018.11.127

[5] Charlton RL , Rossi‐Bergmann B , Denny PW , Steel PG . Repurposing as a strategy for the discovery of new anti‐leishmanials: the‐state‐of‐the‐art. Parasitology. 2018;145(2):219‐236. 28805165 10.1017/S0031182017000993 PMC5964475

[6] Telleria CM . Drug repurposing for cancer therapy. J Cancer Sci Ther. 2012;4(7):ix‐xi. 22984635 10.4172/1948-5956.1000e108 PMC3440183

[7] Senanayake SL . Drug repurposing strategies for COVID‐19. Future Drug Discovery. 2020;2(2). 10.4155/fdd-2020-0010

[8] Shereen MA , Khan S , Kazmi A , Bashir N , Siddique R . COVID‐19 infection: origin, transmission, and characteristics of human coronaviruses. J Adv Res. 2020;24:91‐98. 32257431 10.1016/j.jare.2020.03.005 PMC7113610

[9] Adhikari SP , Meng S , Wu YJ , et al. Epidemiology, causes, clinical manifestation and diagnosis, prevention and control of coronavirus disease (COVID‐19) during the early outbreak period: a scoping review. Infect Dis Poverty. 2020;9(1):29. 32183901 10.1186/s40249-020-00646-x PMC7079521

[10] Zhu N , Zhang D , Wang W , et al. A novel coronavirus from patients with pneumonia in China, 2019. N Engl J Med. 2020;382(8):727‐733. 31978945 10.1056/NEJMoa2001017 PMC7092803

[11] Xu H , Zhong L , Deng J , et al. High expression of ACE2 receptor of 2019‐nCoV on the epithelial cells of oral mucosa. Int J Oral Sci. 2020;12(1):8. 10.1038/s41368-020-0074-x 32094336 PMC7039956

[12] Toljan K . Letter to the editor regarding the viewpoint “evidence of the COVID‐19 virus targeting the CNS: tissue distribution, host‐virus interaction, and proposed neurotropic mechanism”. ACS Chem Nerosci. 2020;11(8):1192‐1194. 10.1021/acschemneuro.0c00174 32233443

[13] Sanders JM , Monogue ML , Jodlowski TZ , Cutrell JB . Pharmacologic treatments for coronavirus disease 2019 (COVID‐19): a review. JAMA. 2020;323(18):1824‐1836. 32282022 10.1001/jama.2020.6019

[14] Arshad U , Pertinez H , Box H , et al. Prioritization of anti‐SARS‐CoV‐2 drug repurposing opportunities based on plasma and target site concentrations derived from their established human pharmacokinetics. Clin Pharmacol Ther. 2020;108(4):775‐790. 10.1002/cpt.1909 32438446 PMC7280633

[15] Wilson CM . In: Long SS , ed. 296 ‐ Antiparasitic Agents, in Principles and Practice of Pediatric Infectious Diseases. Fourth ed. London: Content Repository Only! 2012:1518‐1545 e3.

[16] DrugBank. *Nitazoxanide*. 2020 [cited 2020 17/04/2020]; Available from: https://www.drugbank.ca/drugs/DB00507

[17] Keiser J , Utzinger J . Chapter 8 ‐ The Drugs We Have and the Drugs We Need Against Major Helminth Infections. In: Zhou X‐N et al., eds. Advances in Parasitology. Academic Press; 2010:197‐230. 10.1016/S0065-308X(10)73008-6 20627144

[18] Wang M , Cao R , Zhang L , et al. Remdesivir and chloroquine effectively inhibit the recently emerged novel coronavirus (2019‐nCoV) in vitro. Cell Res. 2020;30(3):269‐271. 32020029 10.1038/s41422-020-0282-0 PMC7054408

[19] Cao J , Forrest JC , Zhang X . A screen of the NIH clinical collection small molecule library identifies potential anti‐coronavirus drugs. Antiviral Res. 2015;114:1‐10. 25451075 10.1016/j.antiviral.2014.11.010 PMC7113785

[20] OpenData Portal . *Nitazoxanide*. 2020 [10/09/2020]; Available from: https://opendata.ncats.nih.gov/covid19/databrowser?q=Nitazoxanide

[21] Bobrowski T , Chen L , Eastman RT , et al. Discovery of synergistic and antagonistic drug combinations against SARS‐CoV‐2 in vitro. bioRxiv: the preprint server for biology. 2020. 10.1101/2020.06.29.178889 PMC7834738 33333292

[22] Rossignol JF , El‐Gohary YM . Nitazoxanide in the treatment of viral gastroenteritis: a randomized double‐blind placebo‐controlled clinical trial. Aliment Pharmacol Ther. 2006;24(10):1423‐1430. 17081163 10.1111/j.1365-2036.2006.03128.x

[23] Korba BE , Elazar M , Lui P , Rossignol JF , Glenn JS . Potential for hepatitis C virus resistance to nitazoxanide or tizoxanide. Antimicrob Agents Chemother. 2008;52(11):4069‐4071. 18710916 10.1128/AAC.00078-08 PMC2573111

[24] Korba BE , Montero AB , Farrar K , et al. Nitazoxanide, tizoxanide and other thiazolides are potent inhibitors of hepatitis B virus and hepatitis C virus replication. Antiviral Res. 2008;77(1):56‐63. 17888524 10.1016/j.antiviral.2007.08.005

[25] Dang W , Xu L , Ma B , et al. Nitazoxanide inhibits human norovirus replication and synergizes with ribavirin by activation of cellular antiviral response. Antimicrob Agents Chemother. 2018;62(11):e00707‐e00718. 30104275 10.1128/AAC.00707-18 PMC6201076

[26] Koszalka P , Tilmanis D , Hurt AC . Influenza antivirals currently in late‐phase clinical trial. Influenza Other Respi Viruses. 2017;11(3):240‐246. 10.1111/irv.12446 PMC5410715 28146320

[27] Rossignol J‐F . Nitazoxanide: a first‐in‐class broad‐spectrum antiviral agent. Antiviral Res. 2014;110:94‐103. 25108173 10.1016/j.antiviral.2014.07.014 PMC7113776

[28] OpenData Portal . *Tizoxanide*. 2020 [10/09/2020]; Available from: https://opendata.ncats.nih.gov/covid19/databrowser?q=Tizoxanide

[29] Pizzorno A , Padey B , Terrier O , Rosa‐Calatrava M . Drug repurposing approaches for the treatment of influenza viral infection: reviving old drugs to fight against a Long‐lived enemy. Front Immunol. 2019;10(531):1‐12. 10.3389/fimmu.2019.00531 30941148 PMC6434107

[30] Haffizulla J , Hartman A , Hoppers M , et al. Effect of nitazoxanide in adults and adolescents with acute uncomplicated influenza: a double‐blind, randomised, placebo‐controlled, phase 2b/3 trial. Lancet Infect Dis. 2014;14(7):609‐618. 24852376 10.1016/S1473-3099(14)70717-0 PMC7164783

[31] Clerici M , Trabattoni D , Pacei M , Biasin M , Rossignol J‐F . The anti‐infective nitazoxanide shows strong immumodulating effects (155.21). 2011;186(1 Supplement):155.21‐155.21. https://www.jimmunol.org/content/186/1_Supplement/155.21/tab-article-info

[32] Miner K , Labitzke K , Liu B , et al. Drug repurposing: the anthelmintics niclosamide and nitazoxanide are potent TMEM16A antagonists that fully bronchodilate airways. Front Pharmacol. 2019;10:51‐51. 30837866 10.3389/fphar.2019.00051 PMC6382696

[33] ClinicalTrials.gov. *Clinical Trials* . 2020 [cited 2020 10/09/2020]; Available from: https://clinicaltrials.gov/

[34] Pepperrell T , Pilkington V , Owen A , Wang J , Hill AM . Review of safety and minimum pricing of nitazoxanide for potential treatment of COVID‐19. J Virus Erad. 2020;6(2):52‐60. 32405422 10.1016/S2055-6640(20)30017-0 PMC7332204

[35] Stockis A , Allemon AM , de Bruyn S , Gengler C . Nitazoxanide pharmacokinetics and tolerability in man using single ascending oral doses. Int J Clin Pharmacol Ther. 2002;40(5):213‐220. 12051573 10.5414/cpp40213

[36] Schaefer IM , Padera RF , Solomon IH , et al. In situ detection of SARS‐CoV‐2 in lungs and airways of patients with COVID‐19. Mod Pathol. 2020;33(11):2104‐2114. 10.1038/s41379-020-0595 32561849 PMC7304376

[37] Nepogodiev D , Bhangu A , Glasbey JC , et al. Mortality and pulmonary complications in patients undergoing surgery with perioperative SARS‐CoV‐2 infection: an international cohort study. The Lancet. 2020;396(10243):27‐38. 10.1016/S0140-6736(20)31182-X PMC7259900 32479829

[38] Zhou F , Yu T , du R , et al. Clinical course and risk factors for mortality of adult inpatients with COVID‐19 in Wuhan, China: a retrospective cohort study. The Lancet. 2020;395(10229):1054‐1062. 10.1016/S0140-6736(20)30566-3 PMC7270627 32171076

[39] Trezza CR , Kashuba ADM . Pharmacokinetics of antiretrovirals in genital secretions and anatomic sites of HIV transmission: implications for HIV prevention. Clin Pharmacokinet. 2014;53(7):611‐624. 24859035 10.1007/s40262-014-0148-z PMC4094112

[40] Cottrell ML , Srinivas N , Kashuba ADM . Pharmacokinetics of antiretrovirals in mucosal tissue. Expert Opin Drug Metab Toxicol. 2015;11(6):893‐905. 25797064 10.1517/17425255.2015.1027682 PMC4498566

[41] Broliden K . Innate molecular and anatomic mucosal barriers against HIV infection in the genital tract of HIV‐exposed seronegative individuals. J Infect Dis. 2010;202(Supplement_3):S351‐S355. 20887223 10.1086/655964

[42] Yao X , Ye F , Zhang M , et al. In vitro antiviral activity and projection of optimized dosing design of hydroxychloroquine for the treatment of severe acute respiratory syndrome coronavirus 2 (SARS‐CoV‐2). Clin Infect Dis. 2020;71(15):732‐739. 32150618 10.1093/cid/ciaa237 PMC7108130

[43] Alexander SPH , Armstrong JF , Davenport AP , et al. A rational roadmap for SARS‐CoV‐2/COVID‐19 pharmacotherapeutic research and development: IUPHAR review 29. Br J Pharmacol. 2020;177(21):4942‐4966. 10.1111/bph.15094 32358833 PMC7267163

[44] Venisse N , Peytavin G , Bouchet S , et al. Concerns about pharmacokinetic (PK) and pharmacokinetic‐pharmacodynamic (PK‐PD) studies in the new therapeutic area of COVID‐19 infection. Antiviral Res. 2020;181:104866. 10.1016/j.antiviral.2020.104866 32659293 PMC7351053

[45] Rajoli RKR , Back DJ , Rannard S , et al. Physiologically based pharmacokinetic modelling to inform development of intramuscular long‐acting nanoformulations for HIV. Clin Pharmacokinet. 2015;54(6):639‐650. 25523214 10.1007/s40262-014-0227-1 PMC4450126

[46] Rajoli RKR , Curley P , Chiong J , et al. Predicting drug‐drug interactions between rifampicin and long‐acting cabotegravir and rilpivirine using physiologically based pharmacokinetic modeling. J Infect Dis. 2019;219(11):1735‐1742. 30566691 10.1093/infdis/jiy726 PMC6500558

[47] Fryar CD , Kruszon–Moran D , Gu Q , Ogden CL . Mean body weight, height, waist circumference, and body mass index among adults: United States, 1999–2000 through 2015–2016. National Health Statistics Reports; no 122. Hyattsville, MD: National Center for Health Statistics; 2018. https://www.cdc.gov/nchs/data/nhsr/nhsr122-508.pdf 30707668

[48] Williams, L. R. Reference values for total blood volume and cardiac output in humans. 1994 [cited Access 1994 Accessed: 18/01/2018]; Available from: https://www.osti.gov/biblio/10186900-reference-values-total-blood-volume-cardiac-output-humans

[49] Bosgra S , van Eijkeren J , Bos P , Zeilmaker M , Slob W . An improved model to predict physiologically based model parameters and their inter‐individual variability from anthropometry. Crit Rev Toxicol. 2012;42(9):751‐767. 22954170 10.3109/10408444.2012.709225

[50] Yu LX , Amidon GL . A compartmental absorption and transit model for estimating oral drug absorption. Int J Pharm. 1999;186(2):119‐125. 10486429 10.1016/s0378-5173(99)00147-7

[51] Peters S . Evaluation of a generic physiologically based pharmacokinetic model for Lineshape analysis. Clin Pharmacokinet. 2008;47(4):261‐275. 18336055 10.2165/00003088-200847040-00004

[52] Rodgers T , Leahy D , Rowland M . Physiologically based pharmacokinetic modeling 1: predicting the tissue distribution of moderate‐to‐strong bases. J Pharm Sci. 2005;94(6):1259‐1276. 15858854 10.1002/jps.20322

[53] Rodgers T , Rowland M . Physiologically based pharmacokinetic modelling 2: predicting the tissue distribution of acids, very weak bases, neutrals and zwitterions. J Pharm Sci. 2006;95(6):1238‐1257. 16639716 10.1002/jps.20502

[54] Sun D , Lennernas H , Welage LS , et al. Comparison of human duodenum and Caco‐2 gene expression profiles for 12,000 gene sequences tags and correlation with permeability of 26 drugs. Pharm Res. 2002;19(10):1400‐1416. 12425456 10.1023/a:1020483911355

[55] Gertz M , Harrison A , Houston JB , Galetin A . Prediction of human intestinal first‐pass metabolism of 25 CYP3A substrates from in vitro clearance and permeability data. Drug Metab Dispos. 2010;38(7):1147‐1158. 20368326 10.1124/dmd.110.032649

[56] Marcellin F , Boyer S , Protopopescu C , et al. Determinants of unplanned antiretroviral treatment interruptions among people living with HIV in Yaoundé, Cameroon. J Trop Med Int Health. 2008;13(12):1470‐1478. 10.1111/j.1365-3156.2008.02170.x 19000156

[57] Broekhuysen J , Stockis A , Lins RL , de Graeve J , Rossignol JF . Nitazoxanide: pharmacokinetics and metabolism in man. Int J Clin Pharmacol Ther. 2000;38(8):387‐394. 10984012 10.5414/cpp38387

[58] Belardo G , Cenciarelli O , la Frazia S , Rossignol JF , Santoro MG . Synergistic effect of nitazoxanide with neuraminidase inhibitors against influenza a viruses *In Vitro* . Antimicrob Agents Chemother. 2015;59(2):1061‐1069. 25451059 10.1128/AAC.03947-14 PMC4335909

[59] Giuseppe Belardo SLF , Cenciarelli O , Carta S , Rossignol J‐F , Gabriella SM . Nitazoxanide, a novel potential anti‐influenza drug, acting in synergism with neuraminidase inhibitors. In *IDSA Annual Meeting*. 2011. Boston, MA, USA, Available from: https://idsa.confex.com/idsa/2011/webprogram/Paper31075.html

[60] Chenel M , Bouzom F , Aarons L , Ogungbenro K . Drug‐drug interaction predictions with PBPK models and optimal multiresponse sampling time designs: application to midazolam and a phase I compound. Part 1: Comparison of uniresponse and multiresponse designs using PopDes. J Pharmacokinet Pharmacodyn. 2008;35(6):635‐659. 19130188 10.1007/s10928-008-9104-6

[61] Nyberg J , Bazzoli C , Ogungbenro K , et al. Methods and software tools for design evaluation in population pharmacokinetics‐pharmacodynamics studies. Br J Clin Pharmacol. 2015;79(1):6‐17. 24548174 10.1111/bcp.12352 PMC4294071

[62] Stockis A , de Bruyn S , Gengler C , Rosillon D . Nitazoxanide pharmacokinetics and tolerability in man during 7 days dosing with 0.5 g and 1 g b.i.d. Int J Clin Pharmacol Ther. 2002;40(5):221‐227. 12051574 10.5414/cpp40221

[63] Tilmanis D , van Baalen C , Oh DY , Rossignol JF , Hurt AC . The susceptibility of circulating human influenza viruses to tizoxanide, the active metabolite of nitazoxanide. Antiviral Res. 2017;147:142‐148. 28986103 10.1016/j.antiviral.2017.10.002

[64] McCreary EK , Pogue JM . Coronavirus disease 2019 treatment: a review of early and emerging options. Open Forum Infect Dis. 2019;2020;7(4):ofaa105. 10.1093/ofid/ofaa105 PMC7144823 32284951

[65] Walls AC , Park YJ , Tortorici MA , Wall A , McGuire AT , Veesler D . Structure, function, and antigenicity of the SARS‐CoV‐2 spike glycoprotein. Cell. 2020;181(2):281‐292.e6. 32155444 10.1016/j.cell.2020.02.058 PMC7102599

[66] Taubenberger JK , Morens DM . The pathology of influenza virus infections. Annu Rev Pathol. 2008;3:499‐522. 18039138 10.1146/annurev.pathmechdis.3.121806.154316 PMC2504709

[67] Ranjbar S , Haridas V , Nambu A , et al. Cytoplasmic RNA sensor pathways and nitazoxanide broadly inhibit intracellular *Mycobacterium tuberculosis* growth. iScience. 2019;22:299‐313. 31805434 10.1016/j.isci.2019.11.001 PMC6909047

[68] Boffito M , Back DJ , Flexner C , et al. Towards consensus on correct interpretation of protein binding in plasma and other biological matrices for COVID‐19 therapeutic development. Clin Pharmacol Ther. 2020. 10.1002/cpt.2099 PMC8359231 33113246

[69] Drugs.com. Nitazoxanide. 2020 [cited 2020 18/04/2020]; Available from: https://www.drugs.com/ppa/nitazoxanide.html

[70] Marcelín‐Jiménez G , Contreras‐Zavala L , Maggi‐Castellanos M , Angeles‐Moreno AP , García‐González A . Development of a method by UPLC‐MS/MS for the quantification of tizoxanide in human plasma and its pharmacokinetic application. Bioanalysis. 2012;4(8):909‐917. 22533565 10.4155/bio.12.41

