# Supplementary material for: Dose prediction for repurposing nitazoxanide in SARS‐CoV‐2 treatment or chemoprophylaxis
Source: Br J Clin Pharmacol. 2020 Dec 1;87(4):2078–88. doi: 10.1111/bcp.14619 (PMC8056737; doi:10.1111/bcp.14619)
Supplement: Supplementary file 1 — Table S1 Nitazoxanide 50% and 90% effective concentrations against various influenza virus strains Figure S1 Comparison of simulated and observed plasma concentration‐time curve of tizoxanide (TIZ) at fasted state: (A) 500 mg, (B) 1000 mg, (C) 2000 mg, (D) 3000 mg and (E) 4000 mg Figure S2 Comparison of simulated and observed plasma concentration‐time curve of tizoxanide (TIZ) for single and multiple dosing regimen (where available) at fed state: (A) 500 mg, (B) 1000 mg, (C) 2000 mg, (D) 3000 mg and (E) 4000 mg Figure S3 Predicted plasma and lung concentrations for optimal doses during the fed state at different regimens reaching steady state ‐ (A) 1400 mg BID, (B) 900 mg TID and (C) 700 mg QID. TIZ, tizoxanide, SD, standard deviation, solid red line indicates clinical C max of 1 g single dose at fed state [35], solid green line represents clinical C max of 500 mg single dose [70] at fed state and the dotted red line represents the EC90 of nitazoxanide for SARS‐CoV‐2 [18] [file BCP-87-2078-s001.docx]

## Supplemental Tables

## Table S1

**Table S1** Nitazoxanide 50% and 90% effective concentrations against various influenza virus strains

| **Drug** | **EC_50_ (µM)** | **EC_50_ (ng/ml)** | **EC_90_ (µM)** | **EC_90_ (ng/ml)** | **Influenza virus strain** | **Reference** |
| --- | --- | --- | --- | --- | --- | --- |
| Nitazoxanide | 3.2 | 983 | 26 | 7989 | A/Puerto Rico/8/1934 (H1N1) | [1] |
|  | 1.6 | 491 | 16.3 | 5008 | A/WSN/1933 (H1N1) |  |
|  | 3.2 | 983 | 20.8 | 6391 | A/California/7/2009 (H1N1pdm09) |  |
|  | 1.9 | 583 | 21.1 | 6483 | Oseltamivir-resistant A/Parma/24/2009 (H1N1) |  |
|  | 3.2 | 983 | 26.8 | 8235 | A/Goose/Italy/296246/2003(H1N1) |  |
|  | 1 | 307 | 13 | 3994 | A/Parma/06/2007(H3N2) |  |
| **Average** | **2.4** | **722** | **20.7** | **6350** | **(for nitazoxanide)** |  |
| Tizoxanide | 3.8 | 1000 | 33.9 | 9000 | H1N1-PR8 | [2] |
|  | 1.9 | 500 | 22.6 | 6000 | H1N1-WSN |  |
|  | 1.5 | 400 | 13.5 | 3600 | H1N1-OST-R |  |
|  | 5.7 | 1500 | 26.4 | 7000 | H1N1-A/GO |  |
|  | 3.8 | 1000 | 75.4 | 20000 | H3N2-A/FI |  |
|  | 1.1 | 300 | 26.3 | 7000 | H3N2-AMD-R |  |
|  | 3.4 | 900 | 22.6 | 6000 | FLU-B |  |
| **Average** | **3.0** | **800** | **31.6** | **8371** | **(for tizoxanide)** |  |

1. Belardo, G., et al., *Synergistic Effect of Nitazoxanide with Neuraminidase Inhibitors against Influenza A Viruses <em>In Vitro</em>.* Antimicrobial Agents and Chemotherapy, 2015. **59**(2): p. 1061-1069.

2. Giuseppe Belardo, S.L.F., Orlando Cenciarelli, Stefania Carta, Jean-Francois Rossignol, M. Gabriella Santoro. *Nitazoxanide, a Novel Potential Anti-Influenza Drug, Acting in Synergism with Neuraminidase Inhibitors*. in *IDSA Annual Meeting*. 2011. Boston, MA, USA, Available from: <https://idsa.confex.com/idsa/2011/webprogram/Paper31075.html>.

## Supplemental Figures

## Figure S1


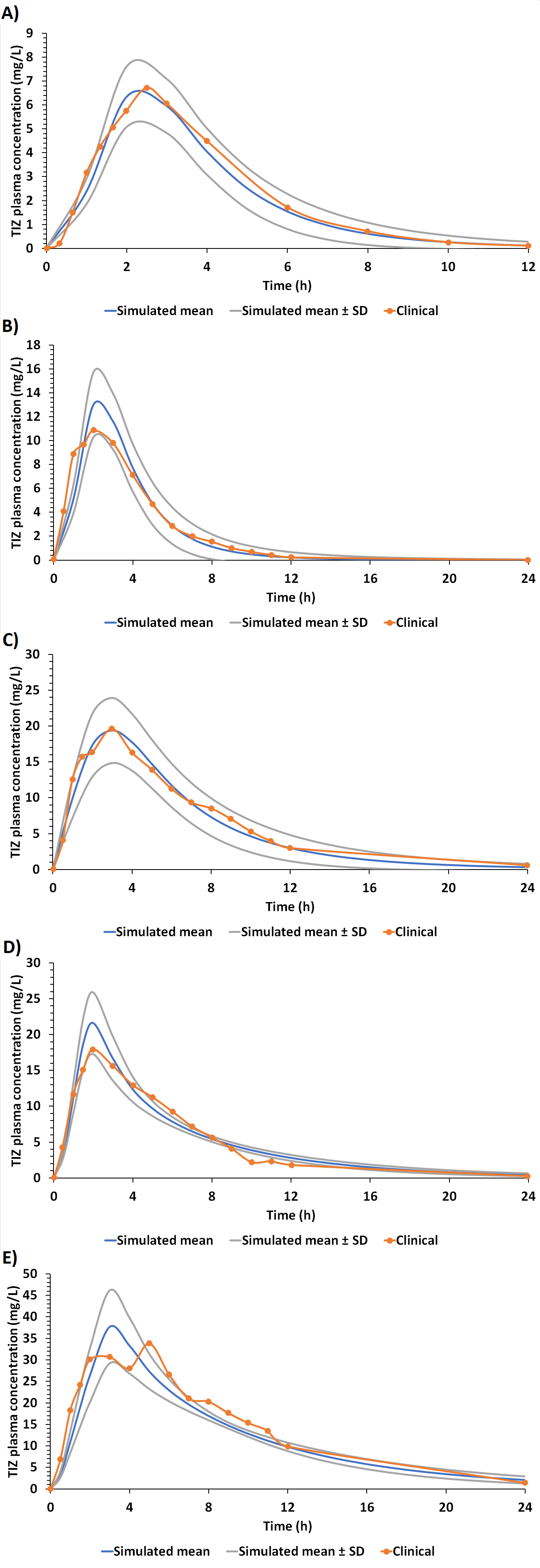


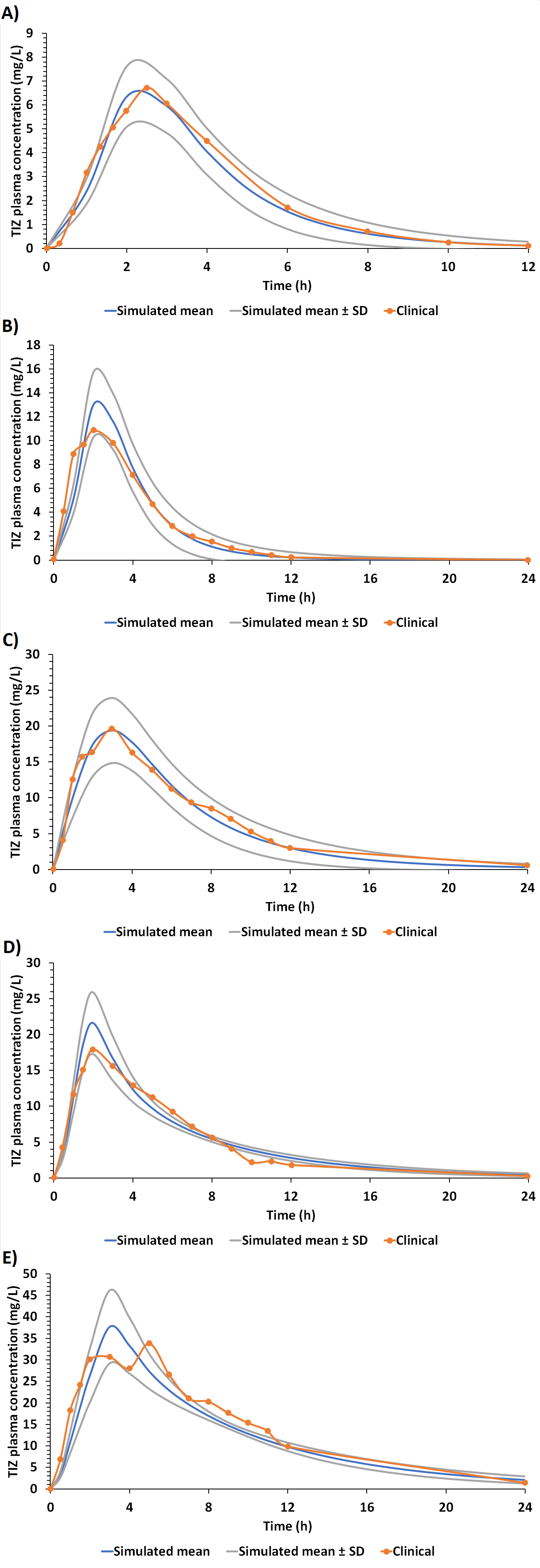


**Figure S1** Comparison of simulated and observed plasma concentration–time curve of tizoxanide (TIZ) at fasted state. (**A**) 500 mg, (**B**) 1000 mg, (**C**) 2000 mg, (**D**) 3000 mg and (**E**) 4000 mg.

## Figure S2


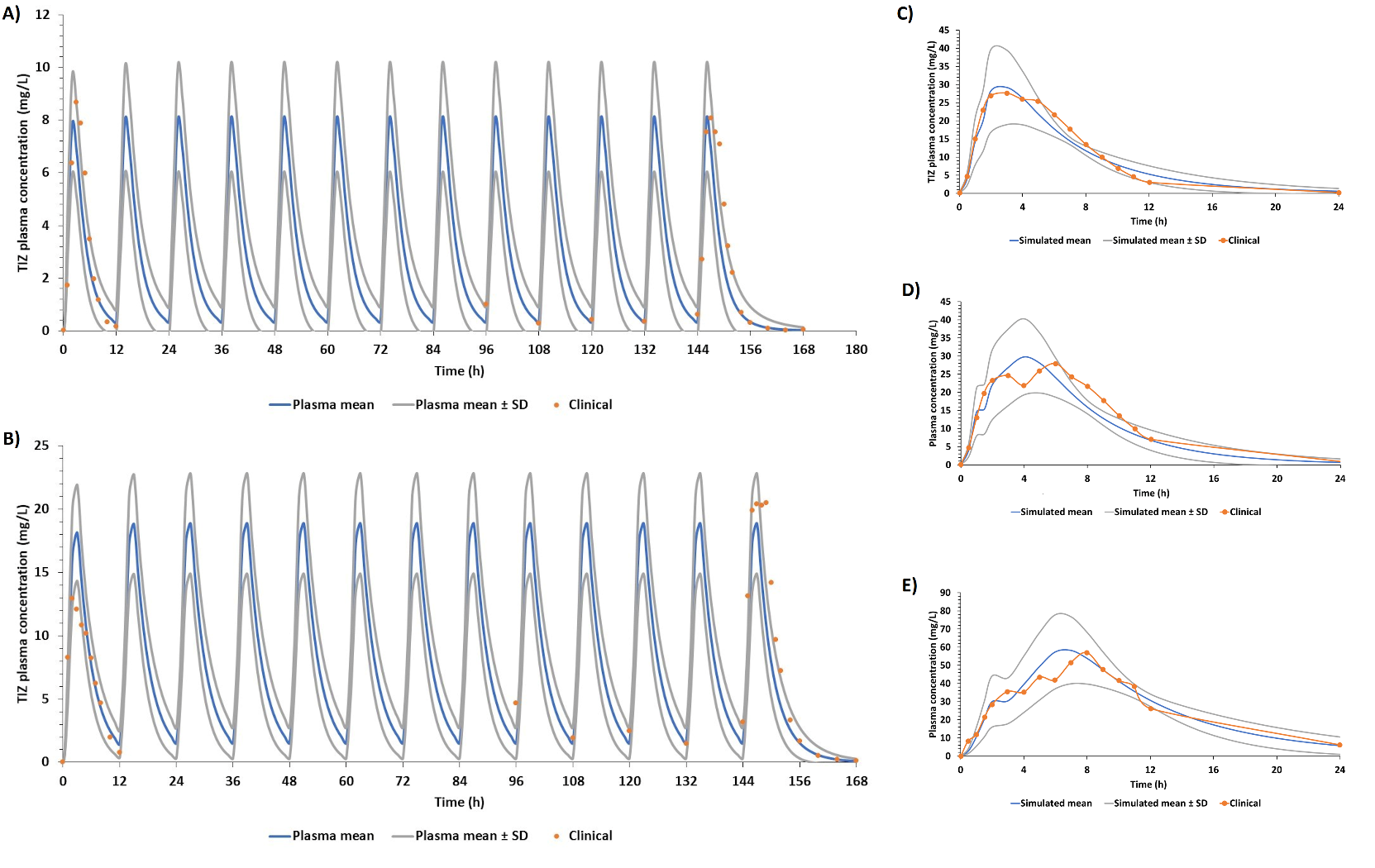


**Figure S2** Comparison of simulated and observed plasma concentration–time curve of tizoxanide (TIZ) for single and multiple dosing regimen (where available) at fed state. (**A**) 500 mg, (**B**) 1000 mg, (**C**) 2000 mg, (**D**) 3000 mg and (**E**) 4000 mg.

**Figure S3**


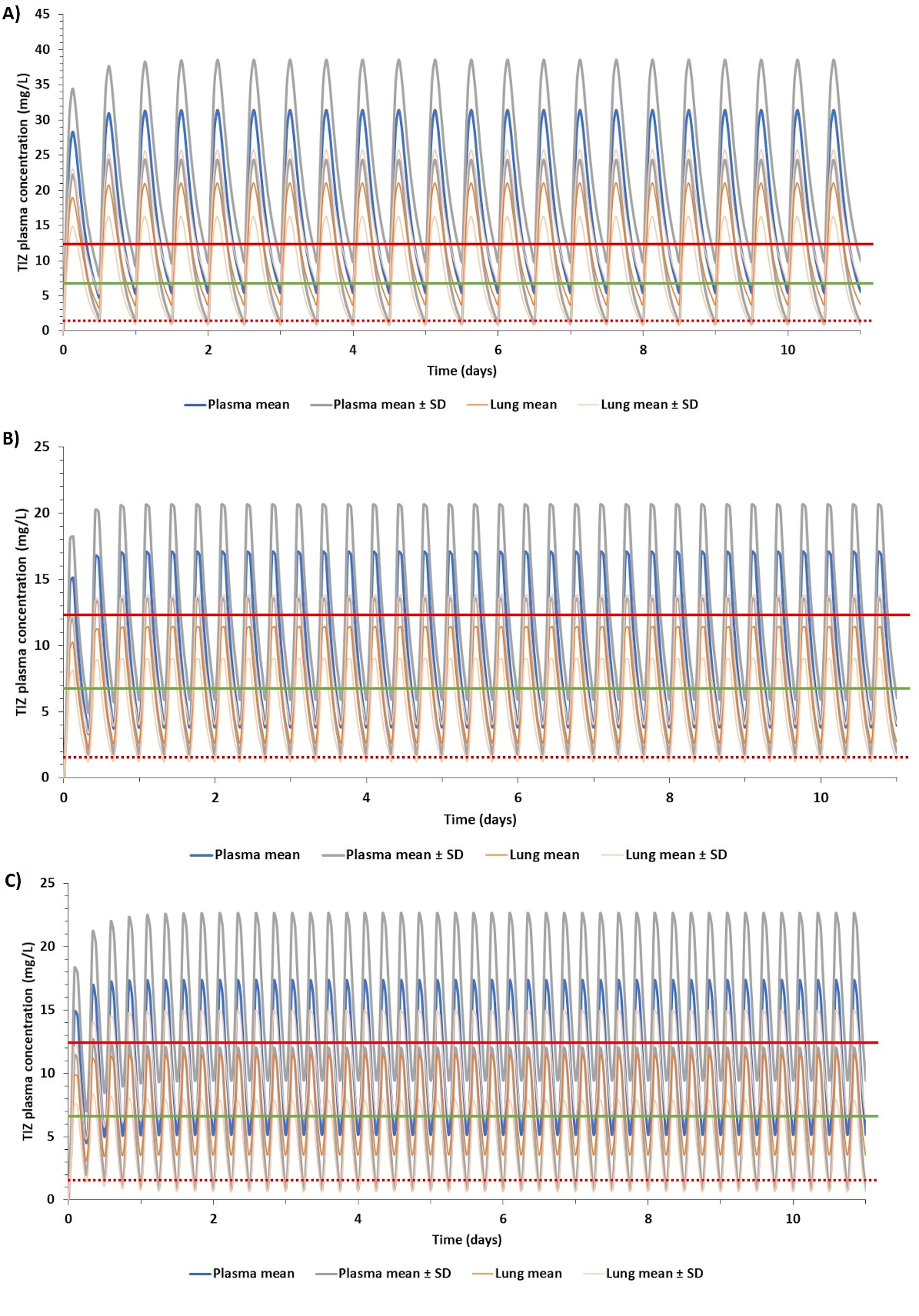


**Figure S3** Predicted plasma and lung concentrations for optimal doses during fasted state at different regimens reaching steady state – (**A**) 2900 mg BID, (**B**) 1600 mg TID and (**C**) 1200 mg QID. TIZ – tizoxanide, SD – standard deviation, solid red line indicates clinical C_max_ of 1 g single dose at fasted state [36], solid green line represents clinical C_max_ of 500 mg single dose [73] at fasted state and the dotted red line represents the EC_90_ of nitazoxanide for SARS-CoV-2 [18].
